# Cluster analysis categorizes five phenotypes of pulmonary tuberculosis

**DOI:** 10.1038/s41598-022-13526-1

**Published:** 2022-06-16

**Authors:** Hyeon-Kyoung Koo, Jinsoo Min, Hyung Woo Kim, Yousang Ko, Jee Youn Oh, Yun-Jeong Jeong, Hyeon Hui Kang, Ji Young Kang, Sung-Soon Lee, Minseok Seo, Edwin K. Silverman, Ju Sang Kim, Jae Seuk Park

**Affiliations:** 1 Division of Pulmonary and Critical Care Medicine, Department of Internal Medicine, Ilsan Paik Hospital, Inje University College of Medicine, Goyang, Republic of Korea; 2 Division of Pulmonary and Critical Care Medicine, Department of Internal Medicine, Daejeon St. Mary’s Hospital, College of Medicine, The Catholic University of Korea, Seoul, Republic of Korea; 3 Division of Pulmonary and Critical Care Medicine, Department of Internal Medicine, Incheon St. Mary’s Hospital, College of Medicine, The Catholic University of Korea, Seoul, Republic of Korea; 4 Division of Pulmonary, Allergy and Critical Care Medicine, Department of Internal Medicine, Kangdong Sacred Heart Hospital, Hallym University College of Medicine, Seoul, Republic of Korea; 5 Division of Pulmonary, Allergy, and Critical Care Medicine, Department of Internal Medicine, Korea University Guro Hospital, Korea University College of Medicine, Seoul, Republic of Korea; 6 Division of Pulmonary and Critical Care Medicine, Department of Internal Medicine, Dongguk University Ilsan Hospital, Dongguk University College of Medicine, Goyang, Republic of Korea; 7 Division of Pulmonary, Critical Care and Sleep Medicine, Department of Internal Medicine, Ulsan University Hospital, University of Ulsan College of Medicine, Ulsan, Republic of Korea; 8 Division of Pulmonary and Critical Care Medicine, Department of Internal Medicine, College of Medicine, Seoul St. Mary’s Hospital, The Catholic University of Korea, Seoul, Republic of Korea; 9 Department of Computer Convergence Software, Korea University, Sejong, Republic of Korea; 10 Channing Division of Network Medicine, Brigham and Women’s Hospital, Boston, MA USA; 11 Division of Pulmonary and Critical Care Medicine, Brigham and Women’s Hospital, Boston, MA USA; 12 Division of Pulmonary Medicine, Department of Internal Medicine, Dankook University College of Medicine, 119 Dandae-ro, Dongnam-gu, Cheonan, 31116 Republic of Korea; 13 Division of Pulmonary and Critical Care Medicine, Department of Internal Medicine, Incheon St. Mary’s Hospital, College of Medicine, The Catholic University of Korea, 56 Dongsu-ro, Bupyeong-gu, Incheon, 21431 Republic of Korea

**Keywords:** Microbiology, Medical research, Risk factors

## Abstract

Tuberculosis (TB) has a heterogeneous phenotype, which makes it challenging to diagnose. Our study aimed to identify TB phenotypes through cluster analysis and compare their initial symptomatic, microbiological and radiographic characteristics. We systemically collected data of notified TB patients notified in Korea and constructed a prospective, observational cohort database. Cluster analysis was performed using K-means clustering, and the variables to be included were determined by correlation network. A total of 4,370 subjects with pulmonary TB were enrolled in the study. Based on the correlation network, age and body mass index (BMI) were selected for the cluster analysis. Five clusters were identified and characterised as follows: (1) middle-aged overweight male dominance, (2) young-aged relatively female dominance without comorbidities, (3) middle-aged underweight male dominance, (4) overweight elderly with comorbidities and (5) underweight elderly with comorbidities. All clusters had distinct demographic and symptomatic characteristics. Initial microbiologic burdens and radiographic features also varied, including the presence of cavities and bilateral infiltration, which reflect TB-related severity. Cluster analysis of age and BMI identified five phenotypes of pulmonary TB with significant differences at initial clinical presentations. Further studies are necessary to validate our results and to assess their clinical implications.

## Introduction

Tuberculosis (TB) remains a major public health problem, and it is a notorious disease owing to heterogeneous phenotypes that make it difficult to diagnose^1^. The spectrum of clinical symptoms of TB varies from asymptomatic cases to severe systemic illness, which is partly understood as a consequence of differences in the microbiologic burden and immunologic status of the host. Because the pathogenesis of this heterogeneity has not been fully established, we are still unable to explain and predict the characteristics of TB patients. Furthermore, a useful classification tool for TB severity has not been developed yet.

To better understand the mechanisms of disease development and progression, it is necessary to define the subtypes of these heterogeneous phenotypes of TB infection. Cluster analysis is an approach of unsupervised machine learning for defining specific groups^2^. This analysis has been employed in diverse disease states, including chronic obstructive pulmonary disease^3^, congestive heart failure^4^, and sepsis^5^. The aim of the present study was to identify specific subtypes in patients with pulmonary TB through cluster analysis. We hypothesised that the demographic features of pulmonary TB patients would affect initial clinical presentation, such as symptoms, microbiological test results and radiographical findings. To select variables for the cluster analysis, we initially searched for demographic features associated with clinical presentation using a correlation network. Thereafter, we used K-means clustering to identify demographic phenotypes of pulmonary TB patients and compared their initial symptoms, along with microbiological and radiographical characteristics among clusters.

## Results

### Baseline characteristics of enrolled patients

Among 5,997 TB patients registered from July 2018 to March 2019, a total of 4,370 subjects with pulmonary TB were finally enrolled after applying the exclusion criteria (Supplemental Figure S1). Their mean age was 58.6 years and 1,623 patients (37.1%) were women. Overall, 2,578 (59.0%) patients had at least one of the prespecified comorbidities, and 2,792 (63.9%) were symptomatic. The most common comorbidity was diabetes (20.7%). In regard to the microbiological features, 1,109 (29.2%) were AFB smear-positive and 2,412 (64.4%) were AFB culture-positive. Regarding radiographic features, 816 (19.1%) had cavities and 1,354 (32.8%) had bilateral disease on chest radiography. Pleural effusion was observed in 151 (3.5%) enrolled participants, and 80 (1.8%) patients had TB disseminated to another non-contiguous organ. Detailed frequencies of each demographic, comorbid, symptomatic, microbiologic and radiographic characteristics are described in Tables 1 and 2.

**Table 1:** Baseline characteristics, socio-economic profiles and comorbidities in the entire study population and among clusters in patients with pulmonary tuberculosis.

	Total (N = 4,370)	Cluster 1 (N = 694)	Cluster 2 (N = 645)	Cluster 3 (N = 1,048)	Cluster 4 (N = 1,106)	Cluster 5 (N = 877)
**Demographics**						
Age, years	58.6 ± 19.7	46.3 ± 12.7	25.8 ± 8.2**	52.7 ± 6.8**	73.6 ± 7.9**	78.3 ± 7.0**
Male sex	2,747 (62.9%)	505 (72.8%)	323 (50.1%)**	795 (75.9%)	665 (60.1%)**	459 (52.3%)**
Female sex	1,623 (37.1%)	189 (27.2%)	322 (49.9%)	253 (24.1%)	441 (39.9%)	418 (47.7%)
Body mass index, kg/m^2^	21.3 ± 3.3	25.9 ± 2.4	20.3 ± 2.2**	19.6 ± 1.9**	23.1 ± 1.6**	17.9 ± 1.9**
**Socio-economic status**						
Medicaid	448 (10.9%)	63 (9.8%)	50 (8.4%)	155 (15.7%)**	74 (7.1%)	106 (12.9%)
Current smoker	979 (22.4%)	208 (30.0%)	182 (28.2%)	402 (38.4%)**	106 (9.6%)**	81 (9.2%)**
Heavy drinker	299 (7.7%)	46 (7.4%)	26 (4.6%)	153 (16.4%)**	41 (4.3%)*	33 (4.2%)*
Previous TB history	748 (17.1%)	106 (15.2%)	61 (9.4%)**	273 (25.9%)**	154 (14.2%)	154 (17.5%)
**Comorbidity**						
Any	2,578 (59.0%)	387 (55.8%)	97 (15.0%)**	541 (51.7%)	871 (78.9%)**	682 (77.8%)**
Diabetes	905 (20.7%)	167 (24.1%)	25 (3.9%)**	211 (20.1%)*	313 (28.3%)	189 (21.6%)
Chronic pulmonary disease	209 (4.8%)	20 (2.9%)	5 (0.8%)**	41 (3.9%)	94 (8.5%)**	49 (5.6%)*
Chronic heart disease	202 (4.6%)	21 (3.0%)	1 (0.2%)**	16 (1.5%)**	92 (8.3%)**	72 (8.2%)**
Chronic liver disease	88 (2.0%)	15 (2.2%)	6 (0.9%)	43 (4.1%)*	17 (1.5%)	78 (0.8%)
Chronic kidney disease	118 (2.7%)	20 (2.9%)	0 (0.0%)**	25 (2.4%)	36 (3.3%)	37 (4.2%)
Neurological disease	371 (8.5%)	23 (3.3%)	8 (1.2%)*	45 (4.3%)	133 (12.0%)**	162 (18.5%)**
Malignancy	431 (9.9%)	47 (6.8%)	8 (1.2%)**	105 (10.0%)*	148 (13.4%)**	123 (14.0%)**
Autoimmune disease	49 (1.1%)	9 (1.3%)	5 (0.8%)	13 (1.2%)	12 (1.1%)	10 (1.1%)
Long-term steroid use	17 (0.4%)	5 (0.7%)	0 (0.0%)	4 (0.4%)	5 (0.5%)	4 (0.3%)
TNF-blocker use	8 (0.2%)	2 (0.3%)	1 (0.2%)	2 (0.2%)	2 (0.2%)	1 (0.1%)
Gastrectomy	47 (1.1%)	3 (0.4%)	0 (0.0%)**	6 (0.6%)	22 (2.0%)*	16 (1.8%)*
Transplant	19 (0.4%)	7 (1.0%)	1 (0.2%)	8 (0.8%)	2 (0.2%)	1 (0.1%)*

TNF, tumour necrosis factor.

*indicate statistical significance of P < 0.05.

**indicate statistical significance of P < 0.01 compared to reference group of cluster 1.

**Table 2:** Clinical, microbiological and radiographic profiles among clusters in patients with pulmonary tuberculosis.

	Total (N = 4,370)	Cluster 1 (N = 694)	Cluster 2 (N = 645)	Cluster 3 (N = 1,048)	Cluster 4 (N = 1,106)	Cluster 5 (N = 877)
**Symptom**						
Asymptomatic	1,578 (36.1%)	308 (44.4%)	276 (42.8%)	408 (38.9%)*	357 (32.3%)**	229 (26.1%)**
Cough or phlegm	1,792 (41.0%)	281 (40.5%)	250 (38.8%)	422 (40.3%)	483 (43.7%)	356 (40.6%)
Dyspnoea	630 (14.4%)	66 (9.5%)	36 (5.6%)**	128 (12.2%)	203 (18.4%)**	197 (22.5%)**
Chest pain	258 (5.9%)	44 (6.3%)	68 (10.5%)**	62 (5.9%)	50 (4.5%)	34 (3.9%)*
Haemoptysis	219 (5.0%)	41 (5.9%)	40 (6.2%)	73 (7.0%)	39 (3.5%)**	26 (3.0%)**
Fever	500 (11.4%)	62 (8.9%)	75 (11.6%)	107 (10.2%)	138 (12.5%)*	118 (13.5%)**
General weakness	215 (4.9%)	8 (1.2%)	9 (1.4%)	49 (4.7%)**	52 (4.7%)**	97 (11.1%)**
Weight loss	342 (7.8%)	19 (2.7%)	58 (9.0%)**	134 (12.8%)**	52 (4.7%)*	79 (9.0%)**
**Microbiologic results**						
AFB smear ( +)	1,109 (29.2%)	134 (22.8%)	125 (22.6%)	315 (35.0%)**	264 (27.4%)*	271 (34.4%)**
AFB culture ( +)	2,412 (64.4%)	325 (56.0%)	343 (62.4%)*	567 (63.7%)**	640 (67.8%)**	537 (68.9%)**
INH mono-resistance	128 (2.9%)	17 (2.4%)	14 (2.2%)	32 (3.0%)	37 (3.4%)	28 (3.2%)
RIF or multidrug resistance	76 (1.7%)	11 (1.6%)	19 (2.9%)*	24 (2.3%)	12 (1.1%)	10 (1.1%)
**Radiographic results**						
Cavity disease	816 (19.1%)	136 (19.9%)	154 (24.3%)*	286 (28.1%)**	109 (10.1%)**	131 (15.3%)*
Bilateral disease	1,354 (32.8%)	148 (22.7%)	138 (22.4%)	380 (38.3%)**	322 (30.9%)**	366 (44.0%)**
**Extrapulmonary involvement**						
Any	228 (5.2%)	39 (5.6%)	40 (6.2%)	38 (3.6%)*	59 (5.3%)	52 (5.9%)
TB pleurisy	151 (3.5%)	29 (4.2%)	20 (3.1%)	24 (2.3%)*	41 (3.7%)	37 (4.2%)
TB lymphadenopathy	26 (0.6%)	4 (0.6%)	12 (1.9%)**	2 (0.2%)	7 (0.6%)	1 (0.1%)
Gastrointestinal TB	20 (0.5%)	1 (0.3%)	5 (0.8%)	6 (0.6%)	5 (0.5%)	2 (0.2%)
Bone/joint TB	26 (0.6%)	3 (0.4%)	0 (0.0%)	4 (0.4%)	8 (0.8%)	10 (1.1%)
CNS TB	6 (0.1%)	0 (0.0%)	2 (0.3%)	2 (0.2%)	1 (0.1%)	1 (0.1%)
Genitourinary TB	3 (0.1%)	1 (0.1%)	1 (0.2%)	0 (0.0%)	0 (0.0%)	1 (0.1%)
Disseminated TB	80 (1.8%)	10 (1.4%)	20 (3.1%)	14 (1.3%)	21 (1.9%)	15 (1.7%)

AFB, acid-fast bacilli; TB, tuberculosis; CNS, central nervous system; INH, isoniazid; RIF, rifampicin.

*indicate statistical significance of P < 0.05.

**indicate statistical significance of P < 0.01 compared to reference group of cluster 1.

### Network analysis

**Figure 1:**
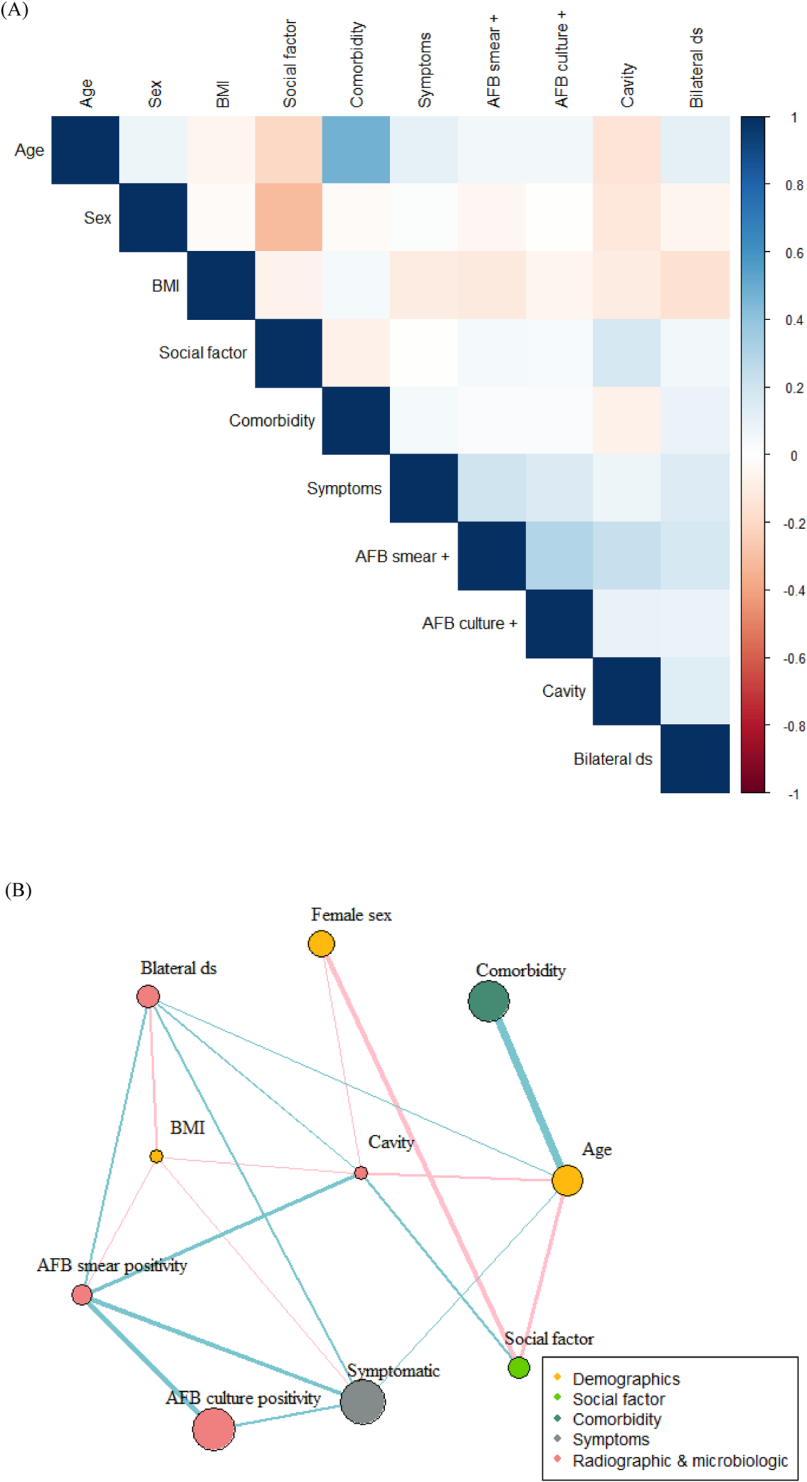
Correlation matrix (**A**) and correlation network (**B**) of variables. (**A**) Pearson correlation between variables was performed. Intensity of colour correlates with strength of their association. Colour of blue indicates positive correlation and that of red for negative correlation. (**B**) Links or edges between nodes indicate the existence of a statistically significant associations (P < 0.05). The thickness of edges correlated with the strength of their association (Pearson’s R coefficient): colour of blue for positive and that of pink for negative correlation. Social factor was defined as being at least one of smoker, drinker, and Medicaid subscriber. BMI, body mass index; AFB, acid-fast bacilli.

To understand the correlations between demographic, social, comorbid, symptomatic, microbiologic and radiographic variables, a correlation matrix (Fig. 1A) and network (Fig. 1B) was built to identify relationships between the variables. Age was positively correlated with the presence of comorbidities and symptoms but negatively associated with the presence of cavities. Body mass index (BMI) was negatively correlated with the presence of symptoms, cavities, and AFB smear positivity.

### Cluster analysis

**Figure 2:**
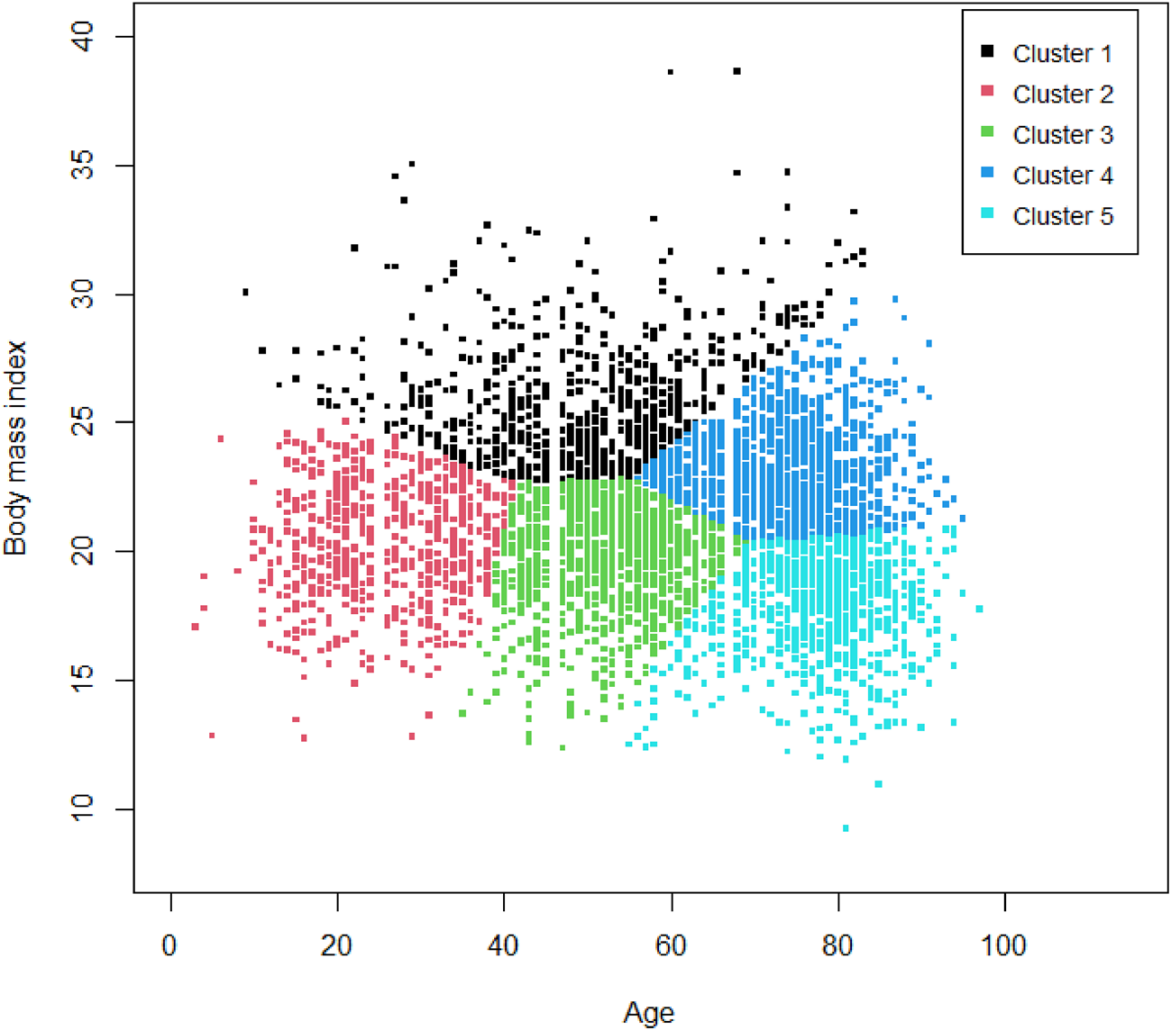
Distribution of each of the five clusters by age and body mass index. X and Y axis means age and body mass index, respectively. Cluster analysis was performed using K-means clustering using kmeans function in the stats package. Two variables—age and BMI—which were considered to contribute to the specific TB phenotype in the correlation network were selected. Colour of cluster indicates each cluster (black: cluster 1, red: cluster 2, green: cluster 3, blue: cluster 4, and sky-blue: cluster 5).

Age and BMI were selected for cluster analysis based on the correlation network. The number of clusters was determined to be five using the silhouette width (Supplemental Figure S2). The distribution of each cluster is shown in Fig. 2. Colour of clusters indicates each cluster (black: cluster 1, red: cluster 2, green: cluster 3, blue: cluster 4, and sky-blue: cluster 5). Adjusted rand index was calculated for 100 randomly selected subsets for validation of clusters, and mean value was 0.80 with standard deviation of 0.18, which suggested good recovery. Random forest was performed to validate the result of clustering with tenfold cross validation. The overall accuracy was 0.990 (P < 0.001) and balanced accuracy for the cluster 1–5 was 0.994, 0.996, 0.992, 0.992, and 0.994, respectively.

**Figure 3:**
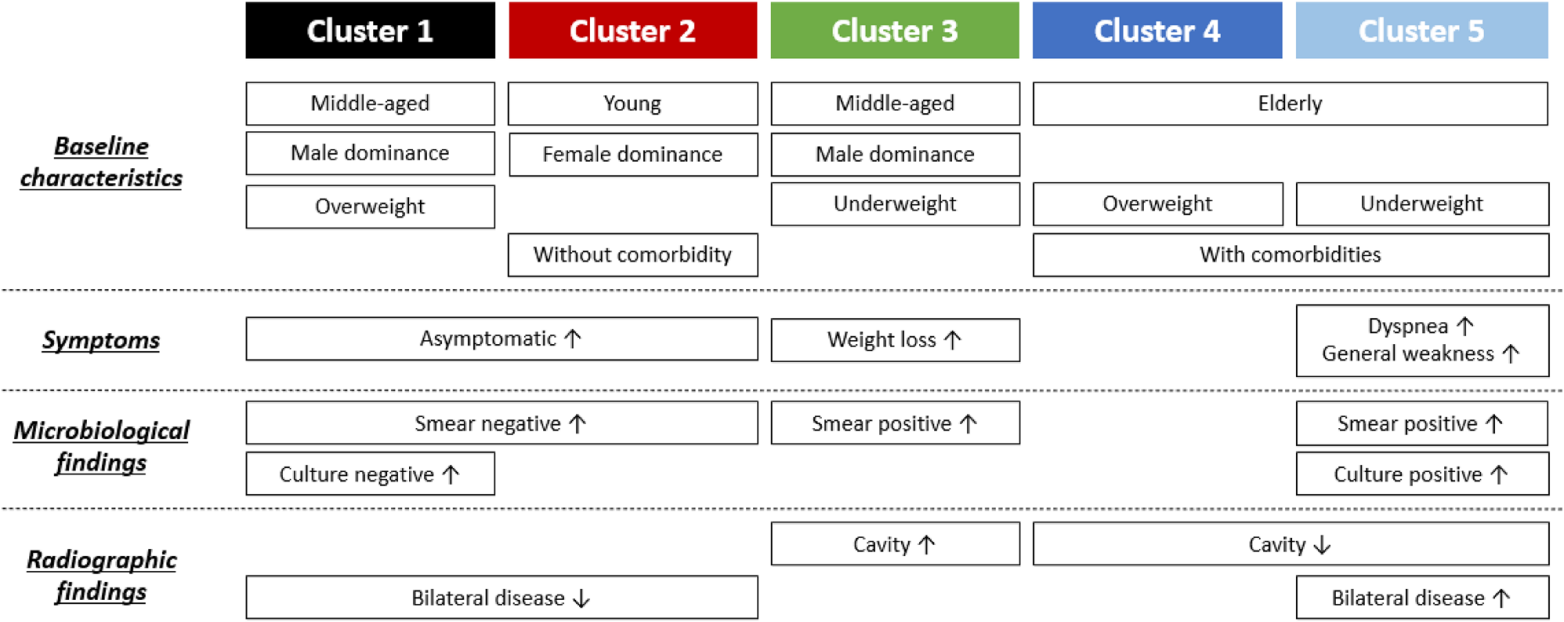
Summary of clinical, symptomatic, microbiological and radiologic characteristics among clusters. AFB, acid-fast bacilli.

**Figure 4:**
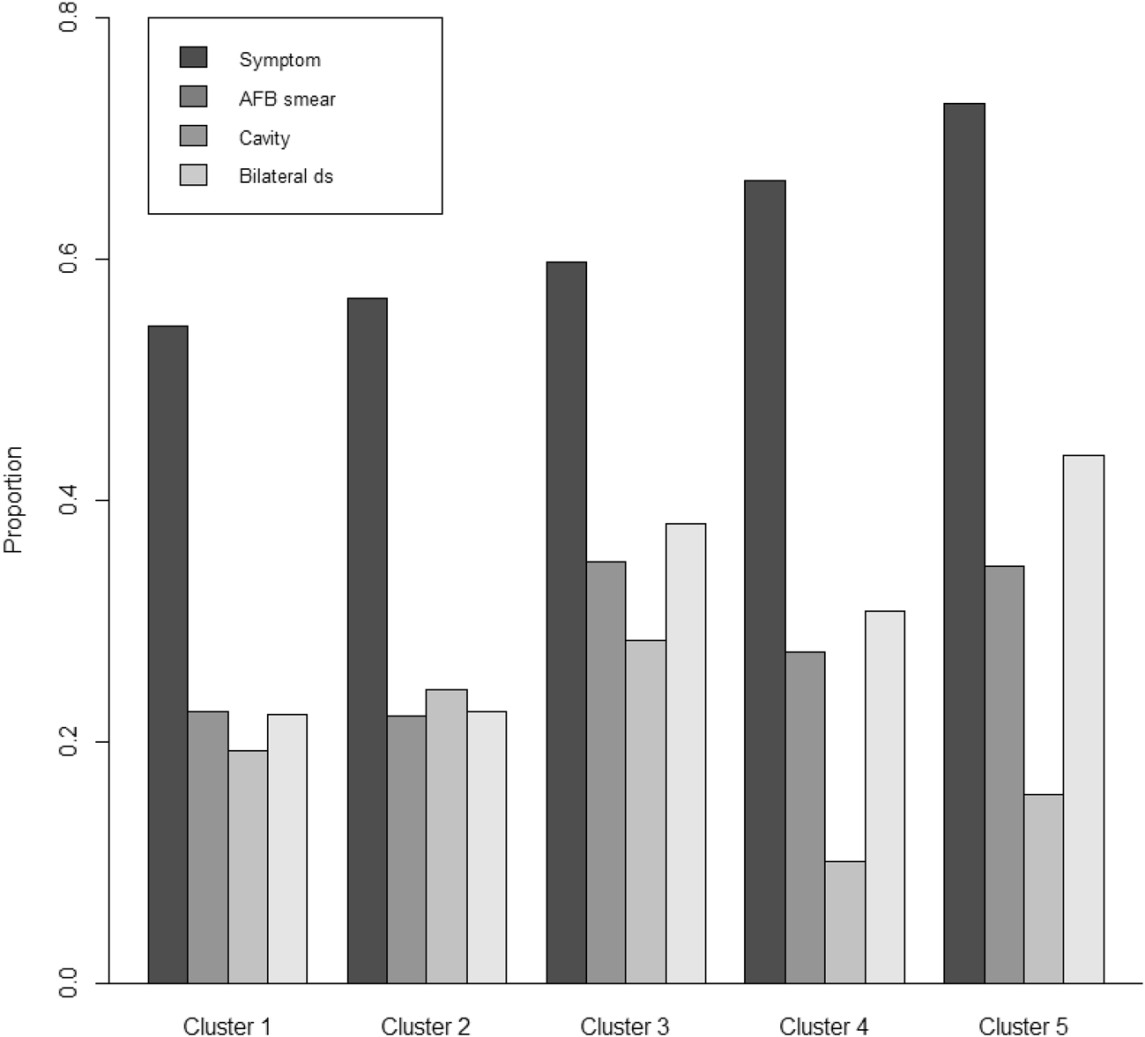
Symptomatic, microbiologic and radiographic differences among five clusters of pulmonary tuberculosis patients. AFB, acid-fast bacilli. Y axis means proportion of symptoms, AFB smear positivity, presence of cavity and bilateral disease according to cluster (X axis).

Based on age and BMI, clusters were designated as follows: (1) middle-aged overweight male dominance, (2) young female dominance without comorbidities, (3) middle-aged underweight male dominance, (4) overweight elderly with comorbidities and (5) underweight elderly with comorbidities (Fig. 3). The symptomatic, microbiological and radiographic differences among the five clusters are plotted and compared in Fig. 4.

#### Cluster 1: middle-aged overweight male dominance

Cluster 1 represented 15.9% (694/4370) of the enrolled participants. The proportion of males (72.8%) and the BMI value (25.9 ± 2.4 kg/m^2^) were the highest among the clusters. Nearly half of the participants (44.4%) did not complain of any TB-related symptoms at initial presentation. Furthermore, positivity for the AFB smear test (22.8%) was the lowest.

#### Cluster 2: young-aged relatively female dominance without comorbidities

Cluster 2 represented 14.7% of the enrolled subjects with the youngest participants, whose mean age was 25.8 years. The proportion of females (50.1%) was the highest among the clusters. The proportions of participants with comorbidities (15.0%) and previous history of TB (94.0%) were the lowest. Compared to the other clusters, chest pain (10.5%) was relatively frequently reported in cluster 2; however, dyspnoea (5.6%) was less common. Proportion of rifampicin- or multidrug-resistant TB (2.9%) was higher than other clusters. The proportions of patients with TB lymphadenopathy (1.9%) and disseminated TB (3.1%) were the highest.

#### Cluster 3: middle-aged underweight male dominance

Cluster 3 represented almost one-quarter of the enrolled participants (24.5%). The proportions of Medicaid patients (15.6%) and heavy drinkers (16.2%) in cluster 3 were the highest, compared to the other clusters. The proportion of patients with chronic liver disease (4.1%) was the highest. Weight loss (12.5%) was most frequently reported in Cluster 3. Previous history of TB (25.9%) was the highest. The proportions of both AFB smear positivity (34.9%) and cavitation on chest radiography (27.6%) were the highest. However, proportion of extrapulmonary involvement (3.6%) was the lowest.

#### Cluster 4: overweight elderly with comorbidities

Cluster 4 also represented nearly one-quarter of the enrolled participants (24.0%). The mean BMI was 23.1 kg/m^2^. Comorbidities were frequent, with the highest proportion of patients with diabetes (28.3%) and chronic pulmonary disease (8.5%). The proportion of patients with cough or sputum (43.7%) was the highest. The proportion of cavitary disease on chest radiography (10.1%) was the lowest.

#### Cluster 5: underweight elderly with comorbidities

Cluster 5 represented 20.1% of the participants who were the most elderly patients with the lowest BMI. The mean age was 78.3 years and the mean BMI was 17.9 kg/m^2^. The proportions of neurologic disorders (18.5%), malignancy (14.0%), chronic heart disease (8.2%) and chronic kidney disease (4.2%) in Cluster 5 were the highest compared to the other clusters. Dyspnoea (22.5%) and general weakness (11.1%) were the most frequently reported symptoms. The proportion of bilateral diseases on chest radiographs (44.0%) and combined bone involvement (1.1%) were highest.

## Discussion

To our knowledge, this is the first study to apply an unsupervised clustering method to categorise TB phenotypes using a large, nationwide cohort database of pulmonary TB. We divided the heterogeneous population into five phenotypes, based on age and BMI, through cluster analysis. We found that patients within each cluster exhibited considerably variable characteristics along with sex, socioeconomic status, comorbidities, symptoms, AFB smear and culture test results and radiographic findings. These findings reconfirm the significant heterogeneity that exists within TB patients and the need for better phenotyping methods.

Advanced age^6,7^ and lower BMI^8,9^ have been positively associated with the development of TB and higher rates of TB-related mortality. Moreover, a negative association between advanced age and the presence of cavities has been reported^10^. We were able to confirm the importance of demographics, including age and BMI, for clinical manifestations and use them for subtyping pulmonary TB patients.

Almost half of the young and middle-aged patients in both clusters 1 and 2 were asymptomatic, which could be regarded as subclinical TB. Our recent study also showed that young and middle-aged patients, aged < 65 years, were more likely to have subclinical TB^11^. In South Korea, where TB incidence is highest among high-income countries, TB screening using chest radiography is regularly performed for adults as part of the health examinations for health insurance members. These health policies have increased the detection of subclinical TB, which could provide valuable opportunities for the early introduction of anti-TB treatment and reduction in TB transmission^12^. Our study revealed that patients in clusters 1 and 2 were more likely to be asymptomatic and have AFB smear negativity and unilateral involvement on chest radiography, which suggested a milder form of pulmonary TB. Symptoms of dyspnoea, fever, general weakness, or weight loss were significantly lower in cluster 1 and 2, except for chest pain. Since patients in these clusters are more likely to be asymptomatic, more attention and thorough investigations are necessary for the diagnosis and treatment of this subgroup.

TB patients in cluster 3 could be considered as a classic phenotype of TB. Their main features were male sex, underweight and social risk factors such as heavy alcohol drinkers and Medicaid beneficiaries. Cluster 3 requires additional attention from clinical and public health perspectives. High frequencies of AFB smear positivity and cavitary disease in this cluster have a greater chance of TB infectivity. Low socio-economic status and prior TB treatment history mean greater possibilities of non-adherence and loss to follow-up^13^. Frequent co-existing chronic liver disease in cluster 3 is an important risk factor for drug-induced liver injury, which may lead to treatment failure or death. Identifying TB patients in cluster 3 and providing patient-centred care should be one of the key interventions of national TB control plans to improve patients’ treatment outcomes and reduce TB transmission.

Although most patients in clusters 4 and 5 were elderly, with a mean age of 70 years, different patterns of prevalent comorbidities had been observed. The proportions of patients with diabetes, chronic pulmonary disease and chronic liver disease were higher in cluster 4, while the proportion of neurological disorders was higher in cluster 5. Its implication needs to be evaluated further. It is unique to observe that elderly patients in cluster 5 had higher proportions of bilateral infiltration on chest radiography and AFB smear positivity. The prominent features of high bilateral infiltration and low cavitation in the chest X-ray in both clusters might indicate impaired host immunity^14^. Previous studies have also revealed that older patients with pulmonary TB have a high rate of middle and lower lobe involvement, which are often accompanied by pleural effusion, mass-like lesions and nodules^15^. A high proportion of smear positivity also increases the chances of TB transmission at home or long-term care facilities in high-income countries. As TB incidence in the elderly population is increasing in Korea, it is crucial to understand their clinical characteristics, which would help physicians to promptly diagnose TB patients^16^.

One of our key findings is that phenotypes of pulmonary TB, such as symptoms, AFB smear positivity and chest X-ray findings, varied among different clusters. These clusters could assist clinicians’ decision to categorise patients during initial assessment, predict TB infectivity and drug-induced liver injury, and prepare patient-centred management. Smear positivity was not correlated with symptoms or cavitation, especially in clusters 4 and 5. Despite the low frequency of symptoms, the presence of cavitation was relatively common in clusters 1 and 2. This variation might be due to the complex pathogenesis of TB, of which we still have limited knowledge. Cluster 2 with young-aged relatively female dominance without comorbidities had high proportions of disseminated TB and TB lymphadenopathy. This might be ascribed to biological differences between male and female, which affects different susceptibility to and phenotypes of TB^17^. In addition, elderly populations in our study had not been grouped into male and female, which could be a result of changes of sex hormones in a trajectory of female lifetime. Further research is also necessary to better adapt future intervention strategies for different sexes. Recent research demonstrated a continuous spectrum of TB infection from latent infection to active disease, which might depend on complex interactions between the host and pathogen^18^. A better understanding of its mechanism would provide more solutions for TB diagnosis and management.

This analysis displays a novel finding regarding subtyping of pulmonary TB; however, there are several limitations that should be acknowledged. First, this study was conducted in Korea, a high-income country with an aging population and low prevalence of human immunodeficiency virus (HIV). If the same analysis was performed in another low-income country with high TB and HIV burden, the clustering results could be inconsistent. To validate our results, further studies in different settings are necessary. Second, our study population was recruited from PPM-participating hospitals in Korea, which oversees approximately 70% of notified TB patients across the country. Exclusion of patients from non-PPM hospitals could limit the generalisability of our results. Third, we did not collect detailed radiographic characteristics such as number or diameter of cavity. Therefore, there are limitations for analyses of such characteristics. Fourth, we could not evaluate how each cluster affected the clinical outcomes. Inferring clinical prognosis based on current results of unsupervised cluster analysis was limited, because the final treatment outcomes could not be assessed. Long-term follow-up of our cohort is required to determine the specific treatment outcomes. Fifth, we could not collect other laboratory findings, such as blood cell counts, c-reactive protein, and interferon-gamma, because of prospective design of our nationwide cohort database, which did not include such important inflammatory biomarkers.

In conclusion, patients within each cluster varied considerably along the measures of symptoms and microbiologic and radiologic findings. Current anti-TB treatment guidelines follow a one-size-fits-all approach, which impedes the improvement of treatment outcomes. Subgrouping TB patients into homogenous phenotypes could provide a stratified medical approach and be a cornerstone for individualising treatment strategies. In order to do so, further studies are necessary to validate our results and assess their clinical implications.

## Methods

### Study design and the Korea TB cohort database

We constructed a prospective, observational cohort database called the ‘Korea TB cohort database’. Data were systematically collected from TB patients who visited the hospitals under the national public–private mix (PPM) TB control project and were notified of the national TB surveillance system. All the notified TB patients were followed-up at regular intervals during the anti-TB treatment, which followed the recommendations of the Korean TB guidelines. For this database, every TB patient notified from the first to the tenth day of each month was consecutively enrolled across the country. During this period, data of participants were collected by TB specialist nurses using the prespecified questionnaire and case report form, and were uploaded into Microsoft Access (Redmond, WA, USA). Then, a regional data manager organised the data gathered from local hospitals every month and forwarded it to a central data manager in a quarterly basis. To improve and maintain data quality, regional and central data managers performed audits and identified missing and erroneous data. For this study, we retrieved data from the Korea TB cohort database from July 2018 to March 2019 and retrospectively analysed them.

### Study setting and participants

Every hospital under the PPM project in Korea participated in this study. The Republic of Korea, a country with an intermediate-TB burden, has a high rate of TB incidence among other high-income countries. The national PPM TB control project was initiated in 2009 and expanded nationwide in 2011^19^. There are more than 210 TB specialist nurses at 127 PPM hospitals and 236 public health officials at 254 public health centres across the country. Approximately 70.7% of newly notified TB patients in Korea were treated at PPM hospitals in 2018. The main inclusion criterion for this study was pulmonary TB. TB patients with extrapulmonary involvement were excluded. People living with HIV were also excluded, since prevalence of HIV infection is low in Korea.

### Variables

We recorded baseline characteristics such as age, sex, BMI, smoking and alcohol history and co-existing comorbidities, such as diabetes, chronic lung disease, chronic heart disease, chronic liver disease, chronic kidney disease, chronic neurologic disorder, any kind of malignancy, autoimmune disease and long-term steroid use. Symptoms related to TB infection, including cough or phlegm, chest pain, dyspnoea, haemoptysis, fever, general weakness and weight loss were collected. Prior history of anti-TB treatment and sites of TB involvement were also recorded. Disseminated TB was defined as tuberculosis infection involving 2 or more non-contiguous organs and also includes military TB. Furthermore, results of microbiological and radiographic tests, such as sputum acid-fast bacilli (AFB) smear and culture tests, and the presence of cavitary and bilateral disease, were also collected. All data were coded as bivariate variables, except age and BMI, which were coded as continuous variables.

### Statistical analysis

The patients’ characteristics are presented as mean and standard deviation for continuous variables and as relative frequencies for categorical variables. Means were compared using a *t*-test or analysis of variance (ANOVA) and categorical variables were compared using a Chi-squared test or Fisher’s exact test. For correlation matrix, Pearson correlation between variables was performed using cor function in the stats package. Correlation matrix was drawn using the corrplot package. Correlation network is based on correlations of network nodes which can be applied to high-dimensional dataset. In the correlation network, each variable was represented as a specific node: the diameter of the node was proportional to the prevalence of the variables, and the colour of the node reflected characteristics to which these variables were included: demographics (age, sex and BMI), social factors (current smoking status, heavy alcohol intake and under Medicaid support), comorbidities (diabetes, chronic lung disease, chronic heart disease, chronic liver disease, autoimmune disease and long-term steroid usage), symptoms (cough/phlegm, dyspnoea, chest pain, haemoptysis, fever, general weakness and weight loss) and microbiologic and radiographic features (AFB smear/culture positivity, presence of cavitary and bilateral disease). Links or edges between nodes indicate the existence of a statistically significant pairwise correlation (P < 0.05). The thickness of the edges correlated with the strength of their association (Pearson’s R coefficient): blue colour for positive and pink for negative correlation. The igraph package was used to visualise the correlation networks.

Clustering analysis is unsupervised machine learning process of partitioning data into subsets according to distance measured by various means. K-means algorithm is one of a type of clustering that identifies K number of centroids, and allocates every data point to the nearest cluster iteratively based on features provided, while keeping the centroids as small as possible. Cluster analysis was performed by K-means clustering using kmeans function in the stats package with two variables: age and BMI, which were considered to contribute to the specific TB phenotype in the correlation network. Clinical, radiographic and microbiological characteristics were compared. Continuous variables were scaled using a standard scale. The number of clusters was selected based on the silhouette width. The silhouette value reflects how similar to its own cluster (cohesion) compared to other clusters (separation). The silhouette width is based on the pairwise difference between and within the cluster distances to validate the clustering performance. Higher value indicates well matched to its own cluster and poorly matched to neighbouring clusters. Distance for silhouette width was measured using the cluster package. The optimal number of clusters is defined when this index reaches its maximum^20^. For the validation of clusters, adjusted rand index was calculated which measures the correspondence between two partitions of the data using 100 subsets of random sampling with 50% sample size. Lastly, random forest was performed to validate the result of clustering using variables of age and BMI, as tenfold cross validation without fine-tunning. All statistical analyses were performed using the R software (version 3.6.0).

### Ethic approval and consent to participate

The Korea Disease Control and Prevention Agency has the authority to hold and analyze surveillance data for public health and research purpose. The study was conducted in accordance with the principles of the Declaration of Helsinki. The Institutional Review Board of Incheon St. Mary’s Hospital, the Catholic University of Korea approved the study protocol (IRB No. OC21ZNSI0063) and waived the need for informed consent as none of the patients were at risk.

## Supplementary Information

Supplementary Figures.

## Data Availability

The data that support the findings of this study are available from Korea Disease Control and Prevention Agency but restrictions apply to the availability of these data, and so are not publicly available. Data are however available from the authors upon reasonable request and with permission of Korea Disease Control and Prevention Agency.

## Abbreviations

AFB: Acid-fast bacilli
BMI: Body mass index
HIV: Human immunodeficiency virus
KDCA: The Korea Disease Control and Prevention Agency
PPM: Public–private mix
TB: Tuberculosis
