# Author Correction: Cluster analysis categorizes five phenotypes of pulmonary tuberculosis

**DOI:** 10.1038/s41598-023-28621-0

**Published:** 2023-01-25

**Authors:** Hyeon-Kyoung Koo, Jinsoo Min, Hyung Woo Kim, Yousang Ko, Jee Youn Oh, Yun-Jeong Jeong, Hyeon Hui Kang, Ji Young Kang, Sung-Soon Lee, Minseok Seo, Edwin K. Silverman, Ju Sang Kim, Jae Seuk Park

**Affiliations:** 1 Division of Pulmonary and Critical Care Medicine, Department of Internal Medicine, Ilsan Paik Hospital, Inje University College of Medicine, Goyang, Republic of Korea; 2 Division of Pulmonary and Critical Care Medicine, Department of Internal Medicine, Daejeon St. Mary’s Hospital, College of Medicine, The Catholic University of Korea, Seoul, Republic of Korea; 3 Division of Pulmonary and Critical Care Medicine, Department of Internal Medicine, Incheon St. Mary’s Hospital, College of Medicine, The Catholic University of Korea, Seoul, Republic of Korea; 4 Division of Pulmonary, Allergy and Critical Care Medicine, Department of Internal Medicine, Kangdong Sacred Heart Hospital, Hallym University College of Medicine, Seoul, Republic of Korea; 5 Division of Pulmonary, Allergy, and Critical Care Medicine, Department of Internal Medicine, Korea University Guro Hospital, Korea University College of Medicine, Seoul, Republic of Korea; 6 Division of Pulmonary and Critical Care Medicine, Department of Internal Medicine, Dongguk University Ilsan Hospital, Dongguk University College of Medicine, Goyang, Republic of Korea; 7 Division of Pulmonary, Critical Care and Sleep Medicine, Department of Internal Medicine, Ulsan University Hospital, University of Ulsan College of Medicine, Ulsan, Republic of Korea; 8 Division of Pulmonary and Critical Care Medicine, Department of Internal Medicine, College of Medicine, Seoul St. Mary’s Hospital, The Catholic University of Korea, Seoul, Republic of Korea; 9 Department of Computer Convergence Software, Korea University, Sejong, Republic of Korea; 10 Channing Division of Network Medicine, Brigham and Women’s Hospital, Boston, MA USA; 11 Division of Pulmonary and Critical Care Medicine, Brigham and Women’s Hospital, Boston, MA USA; 12 Division of Pulmonary Medicine, Department of Internal Medicine, Dankook University College of Medicine, 119 Dandae-ro, Dongnam-gu, Cheonan, 31116 Republic of Korea; 13 Division of Pulmonary and Critical Care Medicine, Department of Internal Medicine, Incheon St. Mary’s Hospital, College of Medicine, The Catholic University of Korea, 56 Dongsu-ro, Bupyeong-gu, Incheon, 21431 Republic of Korea

Correction to: *Scientific Reports* 10.1038/s41598-022-13526-1, published online 16 June 2022

The original version of this Article contained an error in the Author Information section.

“These authors contributed equally: Hyeon-Kyoung Koo, Jinsoo Min, Ju Sang Kim and Jae Seuk Park.”

now reads:

“These authors contributed equally: Hyeon-Kyoung Koo and Jinsoo Min. These authors jointly supervised this work: Ju Sang Kim and Jae Seuk Park”.

The original Article has been corrected.

