# Supplementary material for: Cluster analysis categorizes five phenotypes of pulmonary tuberculosis
Source: Sci Rep. 2022 Jun 16;12:10084. doi: 10.1038/s41598-022-13526-1 (PMC9203754; doi:10.1038/s41598-022-13526-1)
Supplement: Supplementary file 1 — Supplementary Figures. [file 41598_2022_13526_MOESM1_ESM.docx]

**Supplemental material**

Supplemental Figure S1. Flow chart of participants enrollment


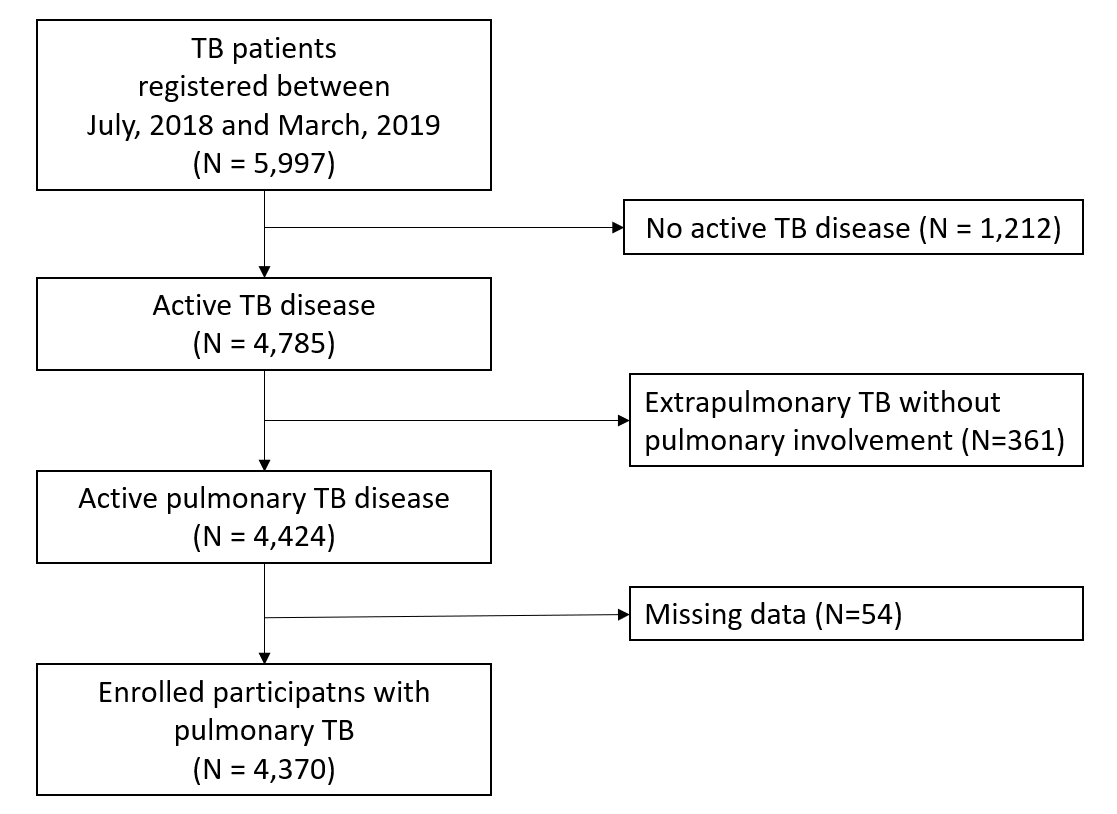


TB, tuberculosis

Supplemental Figure S2. Plots of Silhouette width according to cluster number


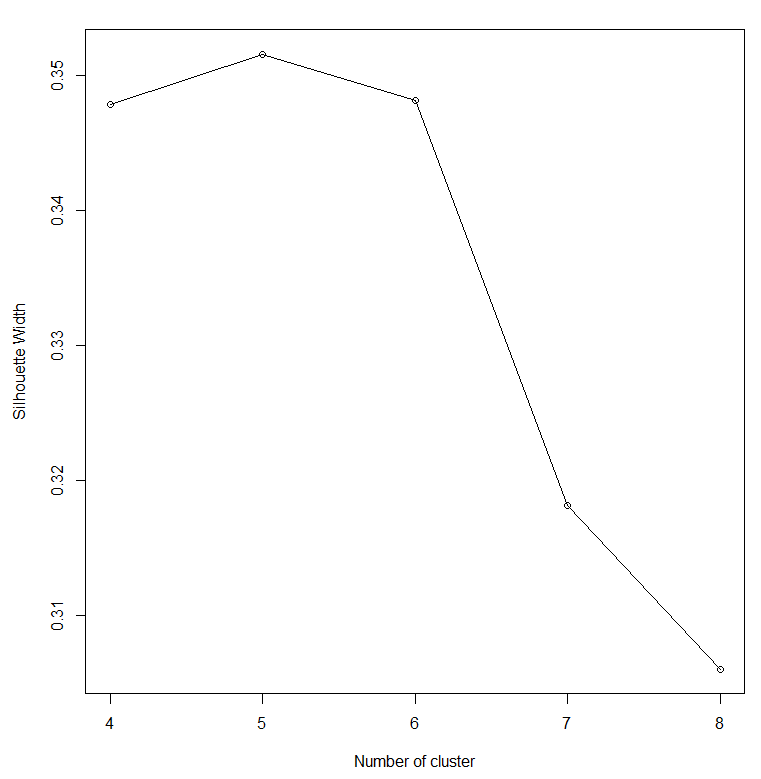


Distance for silhouette width was measured using the cluster package of R software (version 3.6.0)
